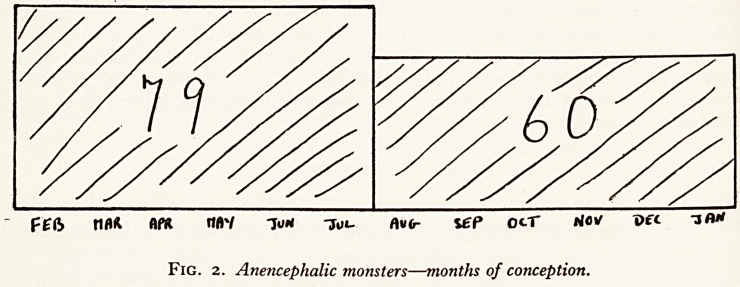# Congenital Malformations

**Published:** 1962-04

**Authors:** N. J. Brown

**Affiliations:** Consultant Pathologist, Southmead Hospital, Bristol


### CONGENITAL MALFORMATIONS.
### Aetiology and Pathology*

BY

N. J. BROWN, M.B., M.R.C.P.

*Consultant Pathologist, Southmead Hospital, Bristol*

Some are born monsters, some achieve monstrosity; others have monsters thrust upon them! I put myself in the last category. When I first went to work at Southmead Hospital some twelve years ago I was no more or less interested in monsters and congenital malformations than in a great many other things. I soon found however, that this problem was indeed thrust upon me. Monsters and malformations came from all directions—so I began to collect them. Some people collect stamps, some people collect shrubs; I began to collect monsters. The collection rapidly grew in size and now contains over a thousand malformations of all kinds.

Southmead Hospital is an interesting place for a pathologist to work in. It not only houses one of the largest maternity hospitals in the country but has, in the same grounds, a general hospital of equal size. The work of the latter tends to give one a sense of proportion and keep one's feet on the ground while the maternity department provides some very valuable material of a specialised nature, including most of the malformations in the collection. Others have come from other hospitals in the group and elsewhere while not a few have come from cases referred by family doctors, having been born at home.

### INCIDENCE OF MALFORMATIONS

For the purposes of this paper I have taken the ten years from August 1951 to August 1961 and analysed some of the results. As will be seen from Table I, during that period 5665 post-mortem examinations were carried out in the hospital, malformations of one

TABLE I

SOUTHMEAD HOSPITAL

1951–1961

| | |
|---|---|
| P.M.s. | 5665 |
| Malformations | 873 |
| Coronary occlusion | 489 |
| Cancer | 894 |
| Baby P.M.s. | 2234 |
| Malformations | 656 |

sort or another being found in 873—about one in every seven. Malformations were almost exactly as common as cancer in our material and were twice as common as coronary thrombosis. About three quarters of the malformations were found in newborn babies, many of them stillborn, but nearly 200 were incidental findings in adults

* A paper read to the Bristol Medico-Chirurgical Society.

who had died from other causes. It should be stated at this point that the definition of malformation used was that of an abnormality of structure due to an error of development. Only anatomical malformations were therefore considered, disorders of function (including fibrocystic disease of the pancreas) being excluded.

The role of congenital malformations as a cause of death in the newly born is illustrated in Table II. If stillbirths and deaths in the first week of life are taken together, approximately one quarter are due to malformations, one quarter to prematurity, one quarter to the various mishaps and diseases of pregnancy and the remaining

TABLE II

Southmead Hospital
Causes of Perinatal Death

| | |
|---|---|
| Congenital Malformations 25% | Prematurity 25% |
| Pre-Eclampsia<br>A.P.H.<br>Placental infarction } 25% | All<br>other<br>causes } 25% |

quarter to all other causes. The *proportion* of deaths from malformations has steadily risen over the last fifty years or so as deaths from other causes have been reduced while the incidence of malformations has remained stubbornly the same and they now constitute the hard core of the problem of perinatal death.

### TYPES OF MALFORMATION

It is quite impossible in a short paper to give a complete account of the variety of malformations found. A few remarks on their occurrence in the various systems of the body must suffice.

By far the largest group consists of malformations of the *central nervous system*. Of these the commonest is anencephaly, which, like the less common iniencephaly and also meningomyelocoele and encephalocoele, represents a failure of proper development of the neural tube. It is constantly associated with very small adrenal glands which lack the normal 'foetal' zone of the cortex. This used to be thought to be due to absence of the pituitary gland but it is now known that some anencephalics have pituitaries (of a sort) and the endocrine abnormality may be due to some lack of nervous control over the hypophysis. Renal tract malformations are commonly associated with anencephaly as are, to a less extent, malformations of the alimentary tract and anterior abdominal wall but abnormalities of the heart and other systems are only rarely associated.

The next commonest malformation of the nervous system is hydrocephalus. This can be due to a variety of causes but the most frequent is the Arnold-Chiari malformation of the medulla and cerebellum which occurs with spinal meningomyelocoele. Associated malformations occur in other systems and a study of these is of some practical importance as this type of malformation is rapidly becoming amenable to surgical treatment. There would be less eagerness to devise surgical operations for this condition if it were frequently combined with other potentially fatal abnormalities. Table III shows the associated anomalies in a series of our cases. There is a frequent association with renal tract malformations but these were mostly of a non-fatal type. There is also a very interesting association with malrotation of the intestine which will be referred to below. The great majority however either had no other malformation or only one of a minor character. It would seem therefore that surgical treatment is not generally contra-indicated on these grounds.

Malformations of the *alimentary tract* are also of great surgical interest. Tracheo-oesophageal fistula and congenital diaphragmatic hernia are treatable surgically if diagnosed early enough and progress has been made in recent years in dealing with the various forms of neonatal intestinal obstruction and of anomalies of the anal canal. Malrotation of the intestine is a not uncommon cause of intestinal obstruction in the newborn but that it can occur without causing any symptoms is shown by the fact that in the present series it was found incidentally at necropsy in no less than three adults, the oldest being 76 years. A congenital type of diaphragmatic hernia with half of the liver, the stomach and most of the small gut in the right side of the chest was found quite unexpectedly in a woman aged 58.

Malformations of the *urinary system* are very common, particularly congenital hydronephrosis and duplication of the ureter. Other fairly frequent findings were "horse-shoe" kidney and polycystic kidney. Enormously enlarged polycystic kidneys, undoubtedly congenital in origin were found in fifteen adults in the series, the oldest case being a woman aged 70. All these cases died of uraemia or of the effects of hypertension and all had congenital cysts in the liver as well. Polycystic kidneys in the newborn were more often associated with other renal tract abnormalities than were those in the adults. There were three cases of complete absence of both kidneys. These all had the typical Potter facies of wide-set slit-like eyes with prominent epicanthic folds and large low-set ears. This facies was not only associated with complete renal agenesis however but was seen also in cases with other gross malformations of the renal tract.

TABLE III

HYDROCEPHALUS WITH MENINGOMYELOCOELE

| Associated major malformations in 101 cases: | |
|---|---|
| No other malformation .. .. .. | 70 |
| Deformity of thorax .. .. .. | 5 |
| Malrotation of intestine .. .. .. | 9 |
| Genu recurvatum .. .. .. .. | 2 |
| Urinary tract malformation .. .. | 16 |
| Bicornuate uterus .. .. .. .. | 2 |

There were 124 cases of *congenital heart disease.* These included almost everything in the book from patent foramen ovale (with, in one case, a paradoxical embolism) to complete absence of the heart. This latter condition was seen twice. Both were acardiac-acephalic monsters with shapeless bodies, rudimentary limbs, rudimentary abdominal organs but no lungs, heart or brain; both were females. One was a quadruplet, the other a twin, the remaining foetuses being normal. This interesting malformation is only seen in multiple pregnancies: the blood is pumped round their bodies in a reverse manner by one of the other babies' hearts through an anastomosis in the placenta or umbilical cord.

Malformations of the *limbs and bones* included polydactyly, syndactyly, talipes and deformities of the spine and ribs. There was one case of sirenomelia—a little mermaid with fused lower limbs and fishy tail—more correctly a 'merboy' since its nuclear sex was male. Of generalised bony disorders there were five cases of achondroplasia and one of fragilitas osseum. Achondroplasiacs are prone to die in the neonatal period from asphyxia, the deformed chest giving no room for the lungs to expand.

### EMBRYOLOGICAL CORRELATION

There were numerous examples of malformations of the *face*, ranging from simple hare lip to several lurid monsters with complete failure of fusion of all the embryonic facial clefts. This leads us on to discuss one approach to the study of the causation of malformations. This is to take a given malformation and then construct an embryological time-table for the development of the affected part. In this way it may be possible to 'date' the origin of the malformation and then a search can be instituted for any possible aetiological factor that might have been operative at that time. Taking hare lip as an example, the upper lip is formed by the fusion of the two maxillary processes with the frontol-nasal process which is known to occur at about the seventh week of development. Failure of these parts to fuse results in a hare lip on one or both sides. One might say therefore that such a malformation has its origin at about the seventh week, something going wrong with development, perhaps only for a matter of hours, at that time. Where multiple malformations are found in diverse systems it is frequently found that the probable time of occurrence is approximately the same for them all and this suggests a common aetiology. Matters do not always work out so neatly however, and this conception of aetiology may be an over-simplified one. It is nevertheless one practical line of approach to a complex problem.

### CAUSES OF MALFORMATION

The factors causing malformations may be considered to fall into two main groups—environmental factors and genetic factors. Fertilisation of the ovum by the sperm is usually said to occur in the outer part of the fallopian tube. The fertilised ovum passes down the tube, dividing as it goes, and becomes embedded in the decidua, usually high up on the posterior wall of the uterus. Here it commences to develop into a baby. As it does so it is dependent for its proper and orderly growth on various factors in its surroundings. If these are abnormal then development may be abnormal and a malformation may result; it is, as it were, a good egg in a bad nest. On the other hand the nest may be perfectly satisfactory but the egg may be bad. This can come about if the fertilised ovum contains a gene which determines a defect. The abnormal gene may be inherited from either parent or both via the sperm or ovum and once present will exert its influence to produce a malformation. It may be able to do this in spite of a perfect developmental environment but there is a good deal of evidence that some genes are weak in expressing themselves and will only produce their effect if encouraged to do so by a bad environment. In other words some malformations may well be due to a combination of genetic and environmental factors.

Some malformations such as achondroplasia are known to have a firm genetic basis, a fact put to practical use in the breeding of dachshunds. So also are some of the inherited disorders of metabolism and sexual anomalies. Others are thought, on fairly strong grounds to have an environmental origin. With the vast majority of malformations, however, we just do not know whether they are genetic or environmental in origin, or both. A little help in sorting out this problem has been provided in the last year or two by the development of methods for the morphological study of chromosomes. This has shown, for example, that some cases of mongolism appear to have a genetic basis, and no doubt other malformations will be intensively studied by this means. To me it seems probable, although this little more than a guess, that environmental factors may well prove to be the more important in the causation of most malformations but only time and further study will provide the answer.

### ENVIRONMENTAL FACTORS

What are these environmental factors which may affect the developing embryo?

1. *Oxygen:* It is easy to see how transient deprivation of oxygen, not severe enough or prolonged enough to cause death, could temporarily inhibit development and produce a malformation. There is abundant experimental evidence that lowering the oxygen concentration in water in which fish eggs are developing will, if carried out at a critical time, produce malformed fish. The human embryo could be temporarily deprived of oxygen by maternal illness, by a fall in maternal blood pressure due to shock or a fainting attack, or by retro-placental haemorrhage in the event of a threatened abortion. Much further study is needed to determine whether or not these factors are important. It is little use asking a mother after the birth of a monster to remember details of the early months of her pregnancy. Only a prospective study of many mothers with careful observation in the early weeks and later follow-up of the outcome will provide the answer.

2. *Temperature:* Similar experiments with fish eggs have shown that changing the water temperature can produce deformities. In the human, it is theoretically possible that a febrile illness might have the same effect but again the importance of this factor has yet to be proved.

3. *Nutrition:* Nutritional defects of vitamins and minerals have been shown to produce malformations in animals but, except possibly in abnormal conditions such as famine, this would not appear to be an important factor in human mothers.

4. *Maternal disease:* It is well known that if a mother contracts rubella in the first three months of pregnancy, she has an increased risk of bearing an infant affected with congenital heart disease, deafness or cataract. The mechanism by which this effect is produced is unknown. Other virus diseases such as influenza have also been incriminated but the evidence against them is less strong.

5. *Maternal age:* This appears to be factor in mongolism, affected children being commoner in older mothers. There does not appear to be any close correlation in other conditions.

6. *Radiation:* Animal experiments have demonstrated that X-rays applied to the developing embryo can induce malformations. There is no reason to suppose that they would not have similar effects on the human embryo and it would certainly seem to be a wise precaution to protect the woman in early pregnancy from excessive doses of radiation. In the case of patients and radiographers the risk is unlikely to be forgotten but with the increasing industrial use of X-rays and other forms of radiation, increasing vigilance will be needed to avoid the inadvertent exposure of young women and expectant mothers to danger. In passing it may be mentioned that X-rays are not only important as an environmental factor. Exposure of the gonads of either sex to radiation may play a part in genetic influence by increasing the mutation rate of genes and increasing the number of abnormal ones.

7. *Drugs.* In the first three months of pregnancy—the vital time when the foundations of malformations are laid—some women take quinine or other abortifacients and fail to produce an abortion. Others are given hormone preparations to encourage the pregnancy to continue. Women are given drugs to make them sleep and drugs to stop them vomiting and tranquillizers to allay their anxiety. Who is to say that all these drugs are harmless to the embryo during this critical stage of its growth?

### SEX OF MONSTERS

Table IV shows an analysis of the sex of the infants affected with malformations. In a few cases the sex was uncertain or not recorded. It will be seen that, in general,

TABLE IV

SEX

| | *F* | *M* | ? |
|---|---|---|---|
| All malformations .. .. | 451 | 200 | 5 |
| Anencephaly .. .. | 104 | 38 | 2 |
| Meningomyelocoele .. | 80 | 42 | — |

malformations occur more than twice as commonly in females as in males. In anencephalic monsters the ratio is nearly three to one in favour of females. The reason for this predominance of malformations in the female is not known. It is all the more remarkable since it is usually stated that more male babies are conceived than females.

### SIDE OF THE BODY AFFECTED

Another interesting and completely unexplained observation has been made from this series of cases. Certain malformations can occur on either side of the body and it seemed to me for some years that left-sided lesions were more common. This impression was confirmed by the finding that of unilateral renal malformations sixty-three were on the left and only thirty-nine on the right. Of congenital diaphragmatic herniae with gross defects in the dome of the diaphragm, nineteen were left-sided and only three right-sided. The numbers were too small for comparison in the other conditions but it certainly seems that renal and diaphragmatic defects, at any rate, are much commoner on the *left* side.

### SEASONAL INCIDENCE

McKeown and Record (1951) found that in Birmingham there was a significant variation in the numbers of anencephalic children born at different times of the year. Figure 1 shows a similar variation in the cases in my series. It is necessary to discard

FIG. 1. *Anencephalic monsters—months of birth.*

FIG. 2. *Anencephalic monsters—months of conception.*

the Gregorian calendar which we now use and, following the example of the Income Tax authorities and other government departments, go back to the old English calendar commencing its year in April. It then becomes clear that far more anencephalics are born in the second half of the "financial" year than in the first half.

Carrying the investigation a stage further the month of conception was worked out for each case, taking into account the degree of maturity of the foetus. When this is similarly plotted (Fig. 2) it appears that more anencephalic monsters are conceived during the six months February to July than in the other half of the year. It has been suggested that this increased rate in babies conceived in the late winter and spring might be associated with the respiratory and virus infections which are prevalent at that time of the year but there is no conclusive evidence on this point.

### GEOGRAPHY

It has often been noted that anencephalic monsters appear to be more numerous in some parts of the world than others and in conclusion I would like to report some attempts to see whether there might be any special local distribution of monsters. Again, anencephalics, the largest single group, were chosen for study and the home addresses of their mothers (the nearest one could get to their place of conception) were plotted on a map of Bristol. Addresses of an equal number of unselected babies were similarly plotted on another map as controls and the two maps were compared. One realises that this is at best a very rough method of investigation since it is based only on cases examined post-mortem. There is however, a very high necropsy rate and there is no reason to believe that the percentage of anencephalics examined was different from the percentage of controls. Also the investigation extends over ten years during which time there was, no doubt, some movement of population.

Everyone who has examined these maps has drawn a different conclusion from them. At first there seemed to be a remarkable tendency for anencephalics to crop up alongside the railway lines; another person tried to correlate them with the old Bristol coalfields—bringing in geology as well as geography! Quite possibly no definite conclusions at all should be drawn from these maps but one feature did seem to stand out and that was the areas where anencephalics did *not* occur. One might have expected that the majority would be found on the new housing estates where the birth rate would be high. That seems definitely not to be so; far more appear in the older parts of the city—Bedminster, around Stapleton Road Station and at Kingswood. Moreover there were no cases at all from Stoke Bishop, Coombe Dingle, Henleaze or Southmead Estate. Virtually no other part of the city was immune.

Further more detailed investigations along these lines might well prove rewarding, particularly examination of the social, family and domestic backgrounds of the parents of monsters but here we begin perhaps to pass out of the realm of the pathologist into wider spheres. The causation of congenital malformations poses many vast and complex problems. Only if it becomes the meeting point of the clinician, pathologist, anatomist, embryologist, geneticist, sociologist and the followers of many diverse scientific disciplines will this fundamental biological riddle be solved.

### REFERENCE

McKeown, T. and Record, R. G., (1951) *Lancet* **1.** 192.

## Figures and Tables

**Fig. 1. f1:**
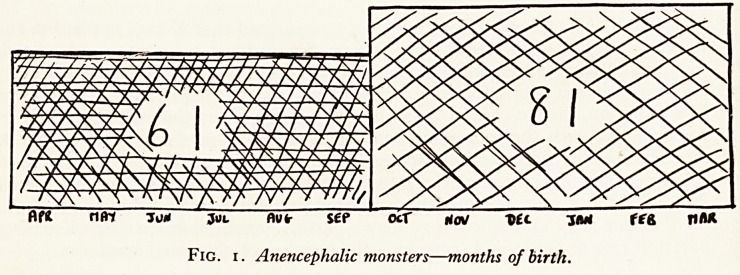


**Fig. 2. f2:**